# Case Report: Sudden cardiac death due to spasm of multiple coronary arteries

**DOI:** 10.3389/fcvm.2025.1647748

**Published:** 2025-08-15

**Authors:** JiaQi Tang, YanDan Xu, MingLei Zhang, ChenChen Wang

**Affiliations:** ^1^ Department of Cardiovascular Medicine, Quzhou KeCheng People’s Hospital, Quzhou, Zhejiang, China; ^2^ Department of Science and Education, Quzhou KeCheng People’s Hospital, Quzhou, Zhejiang, China; ^3^ Department of Pharmacy, Quzhou KeCheng People’s Hospital, Quzhou, Zhejiang, China

**Keywords:** case report, cardiac death, diffuse coronary artery spasm, early diagnosis, standardized drug therapy

## Abstract

Diffuse coronary artery spasm (DMV-CAS) is a serious vascular condition characterized by prolonged narrowing of two or more major coronary arteries or their main branches, leading to significant stenosis or blockage (≥70%). This can result in myocardial ischemia, heart attacks, and dangerous arrhythmias. A 68-year-old male with a four-year history of recurrent angina presented with acute-onset chest tightness, palpitations, and syncope. During transport, the patient experienced prehospital cardiopulmonary arrest, with transient return of spontaneous circulation (ROSC) achieved in the emergency department. Electrocardiographic evaluation revealed atrial fibrillation with rapid ventricular rate, pathological Q waves in the inferior, anterior, and anterior septal territories, along with dynamic ST-T abnormalities, including ST elevation in leads II, III, aVF, and V1-6, and ST depression in leads I and aVL. Emergent coronary angiography identified critical multivessel stenoses, with the most significant narrowing observed in the left anterior descending artery. The diagnosis of DMV-CAS was corroborated through angiographic evidence, demonstrating resolution of the spasm following the administration of intracoronary nitroglycerin (200 μg administered bilaterally to the coronary arteries). Despite the implementation of targeted vasodilator therapy, the patient progressed to refractory cardiogenic shock and succumbed in the intensive care unit 1 h after the procedure. This case underscores the rare and severe cardiovascular implications of DMV-CAS, emphasizing the critical need for early and accurate diagnosis of DMV-CAS and the necessity for standardized pharmacological intervention.

## Introduction

Diffuse Coronary Artery Spasm (DMV-CAS) is a rare but serious condition characterized by severe, reversible spasm affecting two or more major coronary arteries. This condition is not common in clinical practice and, due to its brief duration, is often difficult to diagnose promptly (1). The occurrence of DMV-CAS may be associated with various factors, including the release of endogenous catecholamines, coronary artery spasm after heart transplantation, and cardiac tamponade, among others (2, 3). Current evidence suggests that DMV-CAS has a lower rate of major cardiovascular events than focal coronary artery spasm and an intermediate prognosis (4). In this report, we describe a rare case of DMV-CAS leading to sudden cardiac death. We hope that this case report will further raise awareness among interventional cardiologists about this rare but critical condition.

## Case report

### Clinical summary

**Figure 1:**
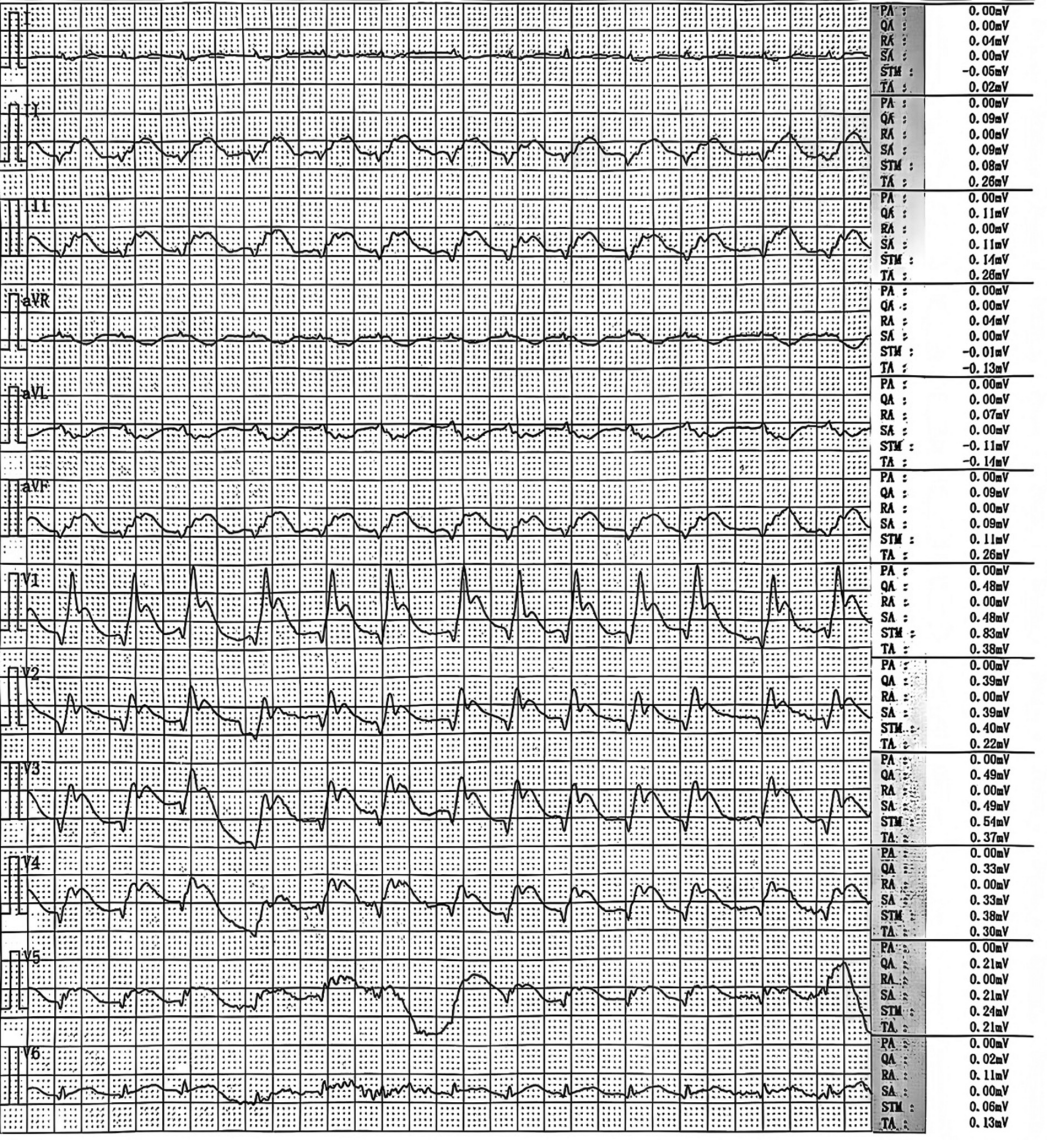
ECG on emergency admission.

A 68-year-old male patient presented with sudden-onset chest tightness and palpitations at home during the early morning hours, with no identifiable precipitating factors. Upon the arrival of emergency medical services (EMS), the patient was found to be unresponsive and in cardiopulmonary arrest. Continuous chest compressions were administered during transport to the hospital. In the emergency department (ED), advanced resuscitation measures were undertaken, including endotracheal intubation, cardiopulmonary resuscitation (CPR), and defibrillation. After resuscitation, an emergency ECG showed atrial fibrillation with a rapid ventricular rate and changes suggesting acute myocardial infarction (Figure 1). These included pathological Q waves in the inferior, anterior, and anteroseptal walls, ST-segment elevation in leads II, III, aVF, and V1-6, and ST-segment depression with T-wave inversion in leads I and aVL. The patient subsequently experienced recurrent cardiac arrests, necessitating ongoing CPR and repeated defibrillation in the ED.

In light of the acute myocardial infarction diagnosis, the patient received dual antiplatelet therapy (aspirin enteric-coated tablet 300 mg and ticagrelor 180 mg), high-intensity statin therapy (atorvastatin 40 mg), and underwent emergent coronary angiography under endotracheal intubation.

### Coronary angiography findings

The initial left coronary angiography revealed no significant stenosis in the left main trunk, an 85% stenosis in the mid-segment of the left anterior descending (LAD) artery, and a 40% stenosis in the mid-segment of the circumflex (LCx) artery, with TIMI 3 flow. Subsequent right coronary angiography encountered technical difficulties due to inadequate catheter engagement. Utilization of a JR4.0 guiding catheter disclosed a slender, elongated proximal right coronary artery (RCA) with approximately 80% stenosis. Administration of intracoronary nitroglycerin (NTG) led to a significant resolution of stenosis, thereby confirming coronary artery spasm as the underlying mechanism.

**Figure 2:**
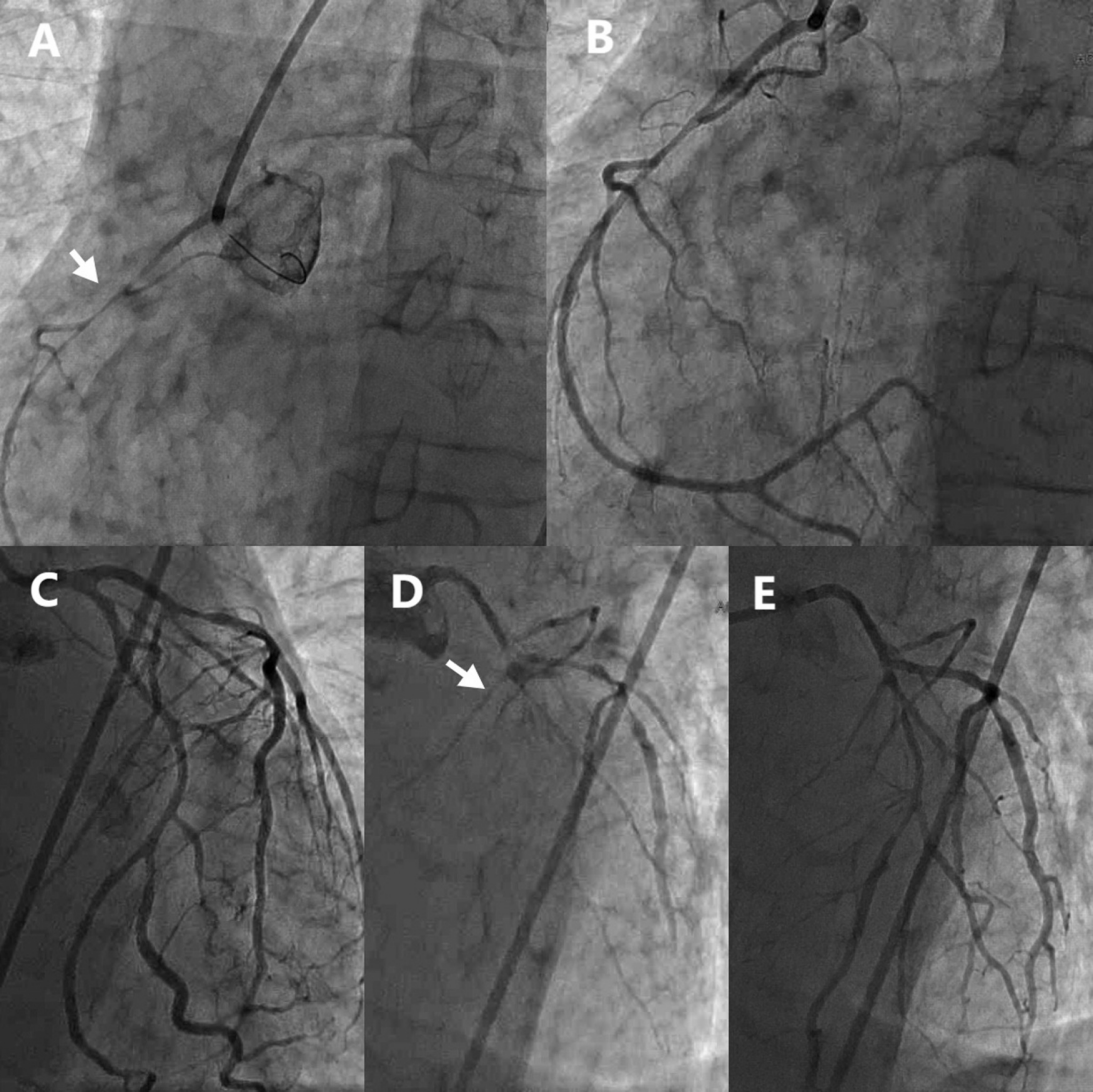
Emergency coronary angiography showed diffuse spasm of multiple coronary arteries. **(A)** RCA before NTG administration. **(B)** RCA after NTG administration. **(C)** Initial LCA imaging findings. **(D)** LCA second imaging results. **(E)** LCA after NTG administration. RCA, Right coronary artery; LCA, Left coronary artery; NTG, Nitroglycerin.

In light of the discordance between the presumed RCA culprit lesion and the initial ECG findings, a repeat left coronary angiography was conducted. This procedure demonstrated a no-reflow phenomenon in the proximal LAD (TIMI 0 flow) and an 85% stenosis in the proximal LCx. The injection of intracoronary NTG (200 µg) into the left coronary system restored full LAD perfusion (TIMI 3 flow), thereby confirming DMV-CAS as the etiology of the LAD lesion. The proximal LCx stenosis improved to 50% post-spasm relief, verifying the coexistence of underlying atherosclerotic plaque (Figure 2).

### Clinical course and past medical history

Following coronary angiography, the patient was transferred to the ICU. Subsequently, he developed progressive bradycardia and hypotension with absent carotid pulses. Immediate CPR was initiated alongside continuous intravenous infusions of epinephrine and norepinephrine. Despite aggressive resuscitation efforts, return of spontaneous circulation (ROSC) was not achieved, and the patient was ultimately declared deceased.

The patient had no documented history of hypertension or diabetes mellitus and denied significant smoking or alcohol use. Neither the patient nor their immediate family members have a history of hypertension, coronary heart disease, or other heart conditions. Three years prior, he underwent coronary angiography for acute myocardial infarction, which revealed: no significant stenosis in the left main trunk, 30% stenosis in the mid-LAD artery, 95% stenosis at the ostium of the first diagonal branch (D1), and 40% stenosis in the mid-LCx artery. Percutaneous transluminal coronary angioplasty (PTCA) was performed on the D1 ostial lesion, resulting in 40% post-PTCA residual stenosis. His postoperative regimen included long-term administration of clopidogrel 75 mg daily, atorvastatin 20 mg daily, and nicorandil 5 mg three times daily. Despite this treatment, he reported persistent anginal symptoms throughout the subsequent three-year period.

## Discussion

Coronary artery spasm (CAS) is characterized by the transient and reversible partial or complete occlusion of the coronary arteries resulting from excessive contraction. This phenomenon significantly contributes to myocardial ischemia and can cause a spectrum of coronary artery disease presentations. These manifestations include asymptomatic myocardial ischemia, exertional angina, Prinzmetal's angina, and acute coronary syndromes such as myocardial infarction or sudden death (5). DMV-CAS is a relatively rare yet severe condition marked by intense and reversible spasms occurring in two or more major coronary arteries. Due to its extensive involvement and transient nature, DMV-CAS is diagnostically challenging and often presents as a complication (1). Although the clinical significance of CAS is frequently underestimated, affected patients face an elevated risk of syncope, severe arrhythmias, and sudden death compared to those with classic angina. Consequently, timely diagnosis is crucial for preventing complications associated with CAS (6).

In this case, the patient initially underwent CAG without evidence of left coronary artery (LCA) spasm. However, the operating surgeon reasoned that isolated RCA spasm could not adequately explain the dynamic, widespread ST-segment changes observed on the ECG. Consequently, repeat left coronary angiography was performed, which revealed transient segmental spasm of the LCA. This finding underscores the importance of maintaining a high clinical suspicion for CAS when discrepancies exist between angiographic findings and ECG evidence of ischemia.

CAS can occur with or without obstructive coronary artery disease (CAD), but it is strongly linked to chest pain and ischemia in patients without obstructive CAD (7). Chest pain in CAS typically occurs at rest, often in the early morning, but can also be triggered by exertion. The pain frequently lasts longer than typical angina and may be accompanied by arrhythmias, diaphoresis, nausea/vomiting, or syncope. CAS-associated ECG changes include ST-segment elevation or depression, U-wave inversion, and T-wave abnormalities (8). The primary risk factors for CAS include age, smoking, hypertension, low-density lipoprotein cholesterol (LDL-C), diabetes, and high-sensitivity C-reactive protein (Hs-CRP) levels (9–11). This patient's onset occurred in the early morning, consistent with the typical timing of CAS onset. However, according to the family's description, there were no notable precipitating factors. It is worth noting that this patient lacked traditional cardiovascular comorbidities (hypertension, hyperglycemia, or dyslipidemia) and other typical risk factors. In such patients presenting with recurrent chest pain but lacking traditional risk factors or triggers, clinicians should maintain a high suspicion for CAS. This warrants considering empiric vasodilator therapy even before a definitive diagnosis is established.

The gold standard for diagnosing CAS is invasive coronary angiography combined with pharmacological provocative testing (e.g., acetylcholine) (12, 13). This can be complemented by intravascular imaging techniques such as optical coherence tomography (OCT) for plaque analysis or intravascular ultrasound (IVUS) to assess myocardial bridging (14). Functional assessments like coronary flow reserve (CFR) and index of microcirculatory resistance (IMR) also provide valuable information (14). Non-invasive approaches include ambulatory ECG monitoring for dynamic ST-segment changes, stress echocardiography, and nuclear imaging (8). Current guidelines recommend provocative testing for intermediate-to-high-risk patients, alongside assessing genetic factors and microvascular function (4). This patient had undergone multiple coronary angiography procedures, yet pharmacological provocative testing was not performed, likely due to safety concerns associated with the test and the patient's informed consent preferences, thereby delaying the definitive diagnosis of CAS at an earlier stage.

The management of CAS requires lifestyle modifications and pharmacological strategies. Lifestyle modifications are essential and include: strict smoking cessation, limiting alcohol and caffeine intake, adopting a low-salt, low-fat diet high in antioxidants, engaging in moderate exercise, and practicing stress management. These changes aim to improve endothelial function. Pharmacologically, calcium channel blockers (e.g., diltiazem) are first-line for long-term prevention. The combination of CCB with long-acting nitrates can have a cumulative effect in the early stages of CAS, reducing the incidence of CAS (15). The combination of statins and CCB helps reduce the occurrence of CAS, particularly in patients with elevated low-density lipoprotein cholesterol (LDL-C) levels (16). Additionally, Rho kinase inhibitors (e.g., fasudil) can effectively prevent acetylcholine-induced CAS and myocardial ischemia (17). α1-adrenergic receptor antagonists are an option for refractory vasospastic angina, particularly if symptoms persist despite treatment with dihydropyridine calcium channel blockers (DHP-CCBs) and long-acting nitrates (18). Excessive use of antiplatelet agents may exacerbate CAS, as reported in the literature (19). For this patient, if CCB and long-acting nitrates had been administered during the initial medical contact, the clinical outcome might have been different.

## Conclusion

This fatal case of DMV-CAS highlights critical diagnostic and therapeutic challenges. Clinicians should maintain a high index of suspicion for DMV-CAS in patients presenting with:

• Sudden cardiac arrest or profound hemodynamic instability with ECG evidence of widespread, dynamic ST-segment changes (e.g., ST-elevation spanning anterior, inferior, and lateral territories) that are discordant with findings of focal obstructive disease on initial angiography.
• Recurrent angina occurring predominantly at rest or in the early morning hours, especially when refractory to standard antianginal therapies and lacking significant fixed coronary stenosis on prior evaluations.

Early recognition is paramount. Crucially, pharmacologic intervention should not be delayed until definitive angiographic confirmation. At the first point of medical contact (e.g., EMS or ED), prompt administration of sublingual or intravenous nitrates and high-dose CCBs is warranted in suspected cases of acute coronary syndrome where spasm is a potential etiology, particularly with the clinical and ECG features described above. This early vasodilator therapy aims to rapidly relieve spasm, restore coronary flow, and potentially avert the catastrophic cascade of malignant arrhythmias and cardiogenic shock observed here.

The patient's rapid demise despite eventual diagnosis underscores the lethal potential of DMV-CAS and the critical importance of proactive, empiric anti-spasm therapy initiated at the earliest suspicion, even before invasive confirmation. Standardized protocols incorporating these early interventions are essential for improving outcomes in this rare but devastating condition.

## Data Availability

The original contributions presented in the study are included in the article/Supplementary Material, further inquiries can be directed to the corresponding author.
